# Evaluating RNA*later*^® ^as a preservative for using near-infrared spectroscopy to predict *Anopheles gambiae *age and species

**DOI:** 10.1186/1475-2875-10-186

**Published:** 2011-07-08

**Authors:** Maggy Sikulu, Kayla M Dowell, Leon E Hugo, Robert A Wirtz, Kristin Michel, Kamaranga HS Peiris, Sarah Moore, Gerry F Killeen, Floyd E Dowell

**Affiliations:** 1 Griffith University, 170 Kessels Road, 4111, QLD, Australia; 2 Queensland Institute of Medical Research, Herston, QLD, Australia; 3 Ifakara Health Institute, Biomedical & Environmental Sciences Thematic Group, Ifakara, Morogoro, United Republic of Tanzania; 4 Centers for Disease Control and Prevention, Atlanta, Georgia, USA; 5 Division of Biology, Kansas State University, Manhattan, Kansas, USA; 6 Biological and Agricultural Engineering Department, Kansas State University, Manhattan, Kansas, USA; 7 USDA ARS, Engineering and Wind Erosion Research Unit, Center for Grain and Animal Health Research, Manhattan, Kansas, USA; 8 Disease Control and Vector Biology Unit, London School of Hygiene and Tropical Medicine, London, UK; 9 Liverpool School of Tropical Medicine, Vector Group, Liverpool, UK

## Abstract

**Background:**

Mosquito age and species identification is a crucial determinant of the efficacy of vector control programmes. Near-infrared spectroscopy (NIRS) has previously been applied successfully to rapidly, non-destructively, and simultaneously determine the age and species of freshly anesthetized African malaria vectors from the *Anopheles gambiae s.l*. species complex: *An. gambiae s. s*. and *Anopheles arabiensis*. However, this has only been achieved on freshly-collected specimens and future applications will require samples to be preserved between field collections and scanning by NIRS. In this study, a sample preservation method (RNA*later*^®^) was evaluated for mosquito age and species identification by NIRS against scans of fresh samples.

**Methods:**

Two strains of *An. gambiae s.s*. (CDC and G3) and two strains of *An. arabiensis *(Dongola, KGB) were reared in the laboratory while the third strain of *An. arabiensis *(Ifakara) was reared in a semi-field system. All mosquitoes were scanned when fresh and rescanned after preservation in RNA*later*^® ^for several weeks. Age and species identification was determined using a cross-validation.

**Results:**

The mean accuracy obtained for predicting the age of young (<7 days) or old (≥ 7 days) of all fresh (n = 633) and all preserved (n = 691) mosquito samples using the cross-validation technique was 83% and 90%, respectively. For species identification, accuracies were 82% for fresh against 80% for RNA*later*^® ^preserved. For both analyses, preserving mosquitoes in RNA*later*^® ^was associated with a highly significant reduction in the likelihood of a misclassification of mosquitoes as young or old using NIRS. Important to note is that the costs for preserving mosquito specimens with RNA*later*^® ^ranges from 3-13 cents per insect depending on the size of the tube used and the number of specimens pooled in one tube.

**Conclusion:**

RNA*later*^® ^can be used to preserve mosquitoes for subsequent scanning and analysis by NIRS to determine their age and species with minimal costs and with accuracy similar to that achieved from fresh insects. Cold storage availability allows samples to be stored longer than a week after field collection. Further study to develop robust calibrations applicable to other strains from diverse ecological settings is recommended.

## Background

Accurate identification of mosquito species is necessary for determining the composition of vector populations, particularly as this changes in the face of differential selective pressure exerted by vector control measures, such as insecticide-treated nets [1,2] or indoor residual sprays [3]. These vector control measures occur mainly where morphologically indistinguishable species co-exist as vector complexes, such as *Anopheles gambiae s. l.*, which dominates malaria transmission in most of Africa. Estimating mosquito age distribution of mosquito populations is also crucial for assessing their capacity to transmit malaria and other pathogens [4,5]. For example, a population dominated by young mosquitoes indicates a successful vector control with ITNs and IRS interventions which reduce longevity and therefore both density and infectiousness [6,7]. Only *anophelines *that are at least eleven days old can transmit malaria parasites due to the period required by the parasites to develop inside the mosquito [8], so even modest reductions of mean survival rates within vector populations can deliver substantive epidemiological impact [4,9-11]. Assessing mosquito population age structure prior and subsequent to control interventions therefore provides a strong indication of the efficacy of that intervention.

Several techniques have been established to estimate the age of *Anopheles *mosquitoes. These include the traditional techniques that involve observation of the morphological changes that occur in the reproductive system of the female mosquitoes to estimate their physiological age [12-16]. Recently biochemical approaches based on age-related changes to the abundance of cuticular hydrocarbons [17] and gene transcription [18-21] have shown considerable promise although they may also be costly [22] and, therefore, have limited application for large-scale ecological or epidemiological studies. Additionally, sibling species identification within the critically important *An. gambiae *complex and the *Anopheles funestus *group from Africa relies almost exclusively upon standard Polymerase Chain Reaction (PCR) [23] and multiplex PCR [24,25] protocols. These protocols are nonetheless time consuming and can be costly in a resource limited area. It is for this reason that in most cases, only a small sample of the population is tested to estimate species distribution in an area. More recently, near-infrared spectroscopy (NIRS) has been developed as a complimentary age grading and species identification tool for Africa's main malaria vectors *An. gambiae s.s*. and *Anopheles arabiensis *mosquitoes [26,27]. NIRS is a rapid, non-destructive tool that can determine age and species of hundreds of mosquitoes per day. No reagents are required and only basic computer skills are needed. This NIRS technique is more cost-effective than PCR after about 7,000 samples have been analysed [26]. However, for mosquitoes, this tool has only been applied to fresh anesthetized samples, limiting its use particularly in large-scale studies where preserving samples collected under widely-dispersed sampling sites is often essential.

Current methods to preserve mosquitoes for DNA extraction or dissection include desiccation and stabilization in various storage buffers. Preservation by desiccation involves complete dehydration of samples over silica gel beads and cotton wool. Silica gel must be kept activated but is widely relied upon particularly in large-scale studies in tropical field settings for preserving mosquitoes prior to DNA and antigen assessment by PCR and ELISA techniques. For subsequent analysis of samples with NIRS however, it is also key that samples are preserved in a way that minimizes chemical degradation. Specifically, NIRS is thought to differentiate sibling species of *An. gambiae s.l*. (*An. gambiae s.s*. and *An. arabiensis*) based on the composition of cuticular hydrocarbons, but may also rely on water content which is known to differ between these two sibling species [28]. Additionally, age-grading of these species depends on gene transcripts [20,21] and change of a range of other bio-molecules including cuticular hydrocarbons [17]. While desiccants are a low-cost alternative to preserve insects for DNA analysis, desiccants must be kept activated and the suitability of insects for dissecting can be poor [29].

Insects are also commonly preserved by suspending in solvents such as ethanol, but studies of this approach for samples to be assessed with NIRS indicate a slight reduction in accuracy relative to scanning fresh samples [30]. Also, solvents leave samples brittle and thus they are difficult to dissect. Other storage procedures used to store biological samples include ultra-cold storage in liquid nitrogen but the costs required for maintenance of liquid nitrogen is prohibitive in most field settings, particularly in resource limited tropical countries.

RNA*later*^® ^(Ambion, Inc., Austin, TX) is an aqueous, non-toxic storage reagent that has been used to preserve mosquito DNA [31] and other samples at room temperature up to one month [32], and indefinite storage time is possible if held at -20°C. Although RNA*later*^® ^is more costly than solvents or desiccants, samples are suitable for DNA extraction and dissection. However, NIRS has not been used to analyse mosquitoes stored in RNA*later*^®^. Since RNA*later*^® ^is currently being used by some researchers to preserve mosquitoes and has some advantages over desiccants and solvents for subsequent DNA analysis and dissection, the objective of this study was to compare the accuracy of NIRS for determining the age and species of freshly anesthetized *An. gambiae s.s*. and *An. arabiensis *to those preserved in RNA*later*^®^.

## Methods

### Mosquito rearing

Three *An. arabiensis *strains (Dongola, KGB, and Ifakara) and two *An. gambiae s.s*. strains (G3 and CDC) were used in this study. The Dongola and KGB *An. arabiensis *strains, were obtained from the Malaria Research and Reference Reagent Resource Center, Atlanta, Georgia, and reared at Kansas State University (KSU), Manhattan, KS, using methods described by Mayagaya *et al *[26]. The Ifakara *An. arabiensis *strain was reared in a semi-field system at the Ifakara Health Institute, Ifakara, Tanzania [33], as described by Sikulu *et al *[27]. The G3 and CDC *An. gambiae s.s*. strains are routinely reared at KSU and CDC Atlanta, respectively. Eight different ages (1, 5, 7, 9, 12, 14, 15 and 21 days) were investigated in this study.

### Scanning and preserving

Mosquitoes were scanned by placing single mosquitoes below a fiber optic probe and collecting reflectance spectra using a LabSpec 5000 spectrometer (ASD Inc, Boulder, CO) as described by Mayagaya *et al *[26]. Live mosquitoes were anesthetized with chloroform before scanning, and then immediately put in 0.5 ml micro-centrifuge tubes before filling the tubes with RNA*later*^®^. The tubes were then stored at -20°C. All mosquitoes remained frozen in RNA*later*^® ^for 1 to 3 weeks before rescanning. Although samples were placed at -20°C, Ambion specifications state that samples are stable for one week at room temperature, and for one month when refrigerated.

### Data analysis

Spectra were analysed using Grams PLS/IQ (Thermo Galactic, Salem, NH). Cross-validation was used to determine the accuracy of predicting mosquito age or species from the fresh or preserved mosquitoes. The cross-validation results were then compared to determine if results from preserved mosquitoes were similar to those obtained from fresh insects. A cross-validation is a leave-one-out self-prediction method where mosquitoes from a set are used to predict the species or age of that same set. The number of factors used in developing models or analyzing results was determined from the Prediction Residual Error Sum of Squares (PRESS) and regression coefficients plots. Generally, all models used 5 to 7 factors. Additional details of the data analysis method have been described [26]. Although the spectrometer measures absorbance from 350-2500 nm, results from only the 500-2350 nm region are reported. The PLS/IQ Coefficients of Determination and the Regression Coefficient plots show that data becomes noisy outside this 500-2350 nm region. NIR spectra at shorter wavelengths are noisy due to low light energy at these wavelengths, and spectra at longer wavelengths are noisy due to low sensor sensitivity.

To test for statistical differences between accuracies obtained for age by NIRS for fresh and preserved specimens, the Mann-Whitney rank test was applied on residual age (difference between actual and predicted age) of fresh and RNA*later*^® ^preserved samples. Binary logistic regression was used to compare the accuracy of classification of samples preserved by the two methods, coding prediction accuracy as a binary dependent variable (correct classification or misclassification of each mosquito into <7 d or ≥7 d old age groups with misclassification as the dependent variable) and including true age category, preservation method, and with or without strain as independent predictors. The analysis was performed on the occurrence of misclassifications resulting from the within-species, cross-validation, analyses of strains maintained in Manhattan, KS, together with strains maintained in the Ifakara semi-field system. The 7 d old age category was used as the reference group for interpretation of the odds ratios (ORs) for the effect of actual age, and the KGB *An. arabiensis *mosquitoes were used as the reference category when interpreting the effect of strain. 95% confidence intervals (CIs) of the results were calculated. Samples aged 21 d were omitted from the logistic regression analyses as this age group was not represented in all strain by age comparisons.

## Results

### Age-grading using the cross-validation technique

Table 1 shows accuracies of predicting mosquito age, or of predicting as young (<7 days) or old (≥7 days) for fresh and preserved samples for all strains reared at all locations. On average, all preserved mosquitoes were classed as young or old with approximately the same accuracy as fresh mosquitoes (*P *= 0.09).

**Table 1:** Accuracy (% correct at each day) of age-grading mosquitoes using near-infrared spectroscopy and a partial least squares regression (PLS) cross-validation

Strains	Preserved/Fresh	No. PLS Factors	n	1d	5d	7d	9d	12d	14d	15d	21d	<7d^1^	≥7d^1^	Total^1^
KGB *An. arabiensis*	Fresh	6	151	89.5	51.4		75.0			100		70.7	88.2	79.5
KGB *An. arabiensis*	Preserved	6	153	92.5	81.6		62.9			97.5		87.2	81.3	84.3
Dongola *An. arabiensis*	Fresh	6	127	87.5	59.3		86.5			97.4		72.5	92.1	84.3
Dongola *An. arabiensis*	Preserved	5	123	95.7	80.0		94.9			100		87.5	97.3	93.5
Ifakara *An. arabiensis*	Fresh	6	205	100	64.4	90.2		100	91.1			78.1	93.9	88.3
Ifakara *An. arabiensis*	Preserved	8	123	100	68.2	90.9		84.8	90.0			94.8	88.3	87.0
G3 *An. gambiae s.s.*	Fresh	5	150	100	33.3		92.1			100		65.3	96.0	80.7
G3 *An. gambiae s.s.*	Preserved	5	145	96.9	68.4		97.4			97.3		81.4	97.3	89.7
CDC *An. gambiae s.s.*	Preserved	5	147	91.7		94.6			97.2		100	91.7	97.3	95.9
Average	Fresh													83.2
Average	Preserved													90.0

n -Number of mosquitoes

^1^Values have been presented as weighted mean.

**Figure 1:**
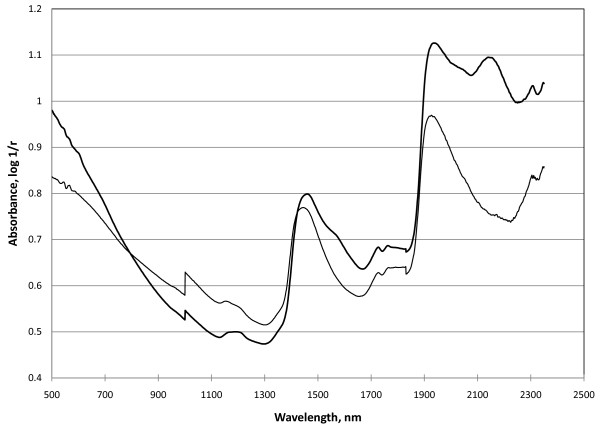
**Near-infrared spectra of fresh (thin line) and preserved (thick line) 15 day old Dongola *An. arabiensis *mosquitoes**.

Figure 1 shows example spectra of fresh and preserved mosquitoes. The differences in absorbance values between fresh and preserved mosquitoes above about 1,500 nm are partly due to the presence of RNA*later*^®^, particularly the absorbance peak at about 2150 nm.

**Figure 2:**
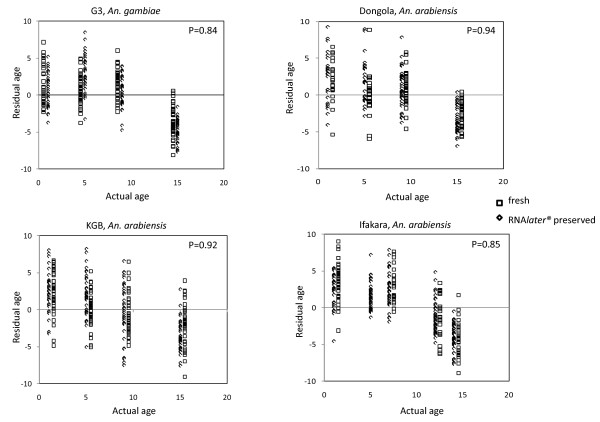
**Age residuals for fresh and RNA*later*^® ^preserved samples of three strains of *An. arabiensis *and one strain of *An. gambiae s.s*. as determined by Mann-Whitney rank test**. No significant differences were observed between medians of residual age for fresh and RNA*later*^® ^preserved specimens for all four strains.

When estimating age as a continuous outcome, inspection of the age prediction residuals for *An. gambiae s.s*. (Figure 2a) and three species of *An. arabiensis *(Figures 2b-2d) indicated that the prediction accuracy was generally to within ± 5 d of actual age and that there was tendency to overestimate the ages of 1-10 d old mosquitoes while the ages of mosquitoes > 10 d tended to be underestimated. However, there was no significant difference between age prediction residuals of fresh and RNA*later*^® ^preserved samples for all species tested (P > 0.05; Mann Whitney U test; Figure 2).

To investigate whether RNA*later*^® ^preservation, strain and actual age affected the success of the predictions of mosquitoes as young or old, multivariate binary logistic regression was performed on the occurrence of misclassification with preservation, strain, and actual age as independent predictor variables. The binary logistic regression model explained a highly significant proportion of the variance in misclassifications (χ^2 ^= 193.76, df = 11, *P *< 0.001) and there was a significant effect of preservation (Wald = 7.30, *P = *0.007) and a highly significant effect of actual age (Wald = 125.12, *P *= *<*0.001); however, the effect of strain was non-significant (Wald = 6.98, *P *= 0.137). The analysis was, therefore, repeated without strain and again showed a significant effect of preservation (Wald = 5.95; *P *= 0.015) and a highly significant effect of actual age (135.61,*P *< 0.001). Mosquitoes preserved in RNA*later*^® ^had a 46% reduction in the likelihood of a misclassification than fresh mosquitoes (OR for misclassification = 0.64, 95% CI = 0.45 - 0.92) when holding actual age at a fixed value. When controlling for the effect of preservation, the probability of a misclassification for most age groups was not different from the 7 d reference age group, except for the 5 d age group, which had a 7 fold greater chance of being misclassified (OR = 6.73, 95% CI = 3.13 - 14.47) and the 15 d age category, which had an 83% reduction in the likelihood of being misclassified (OR = 0.17, 95% CI = 0.05 - 0.59).

### Species identification

*Anopheles arabiensis *and *An. gambiae s.s*. were assigned a value of 1.0 and 2.0, respectively, for the purposes of developing the PLS models. Thus, mosquitoes close to the 1.5 threshold were most likely to be misclassified. When using a cross-validation with Dongola *An. arabiensis *and G3 *An. gambiae s.s.*, there was no added advantage in using fresh or preserved mosquitoes to determine insect species, with classification accuracy about 80% for both species (Table 2).

**Table 2:** Accuracy of determining species of fresh or preserved mosquitoes by near-infrared spectroscopy and a partial least squares (PLS) regression cross-validation

Status	*n*	No. of PLS Factors	Dongola *An. arabiensis*^1 ^Correct, %	G3 *An. gambiae s.s.*^1 ^Correct, %	Average^1 ^Correct, %
Fresh	267	8	82.1	81.3	81.6
Preserved	268	9	76.4	83.4	80.1

n -Number of mosquitoes

^1^Values have been presented as weighted mean.

## Discussion

In this study, RNA*later*^® ^was used to preserve *An. gambiae s.s*. and *An. arabiensis *mosquitoes for subsequent scanning with NIRS to determine their age and species. Age-grading results were generally slightly better when using preserved samples than fresh ones. This may be due to the mosquitoes being more easily and consistently positioned after preserving in RNA*later*^® ^than when scanning fresh. The legs and wings of fresh mosquitoes often contact the NIR probe and thus scatter incident or reflected light, contributing to noise in the spectra and misclassifications. Also, some anesthetized mosquitoes move during scanning and further contribute to noise in the spectra. However, results for species identification were generally similar for both fresh and preserved samples.

In previous studies, NIRS estimated the age and species of fresh anesthetized laboratory-reared, semi-field reared and wild caught *An. gambiae s.s*. and *An. arabiensis *[26,27]. This tool has also earlier been applied successfully to age grade stored-grain pests [34], biting midges [35] and house flies [30] as well as to differentiate between species and subspecies of termites [36]. This rapid and non-destructive technique can generally classify mosquitoes into young and old age groups, and differentiate between morphologically identical *An gambiae s.s*. and *An arabiensis *sibling species of *An. gambiae s.l*. The most recent study on *An. gambiae s.l*. indicated that NIRS could differentiate *An. gambiae s.s*. and *An. arabiensis *sibling species of the *An. gambiae complex *from semi-field and field settings with 89% and 90% accuracy, respectively, and as either young (<7 days) or old (≥7 days) from semi field system with 84% accuracy [27]. In this study, age and species prediction accuracies obtained were consistent with results obtained earlier on fresh samples [26,27]. Since NIRS is a very fast technique, a large number of samples can be scanned in a very short period of time. Therefore, since a more representative sample of the population is analysed when compared to conventional techniques, the 80-90% level of accuracy reported herein and in previous studies may give researchers a better estimate of the true population structure when compared to other methods that may be limited to sample numbers 10 or 100 times smaller than can be used with this NIRS technique.

It was noted that when using the cross-validation technique, the accuracies for classifying very young (1 day) and old (9 and 15 days) was as high as 100% for both fresh and preserved samples. However, 6-8 days old mosquitoes were less accurately predicted as 7 days was nominated as the age defining young and the old ages in this and the previous age classification models [26,27]. This age enables the distinction of female mosquitoes that are more likely to harbour mature parasites (sporozoites) in their salivary glands (≥7 days old) from those that are unlikely to be infectious (<7 days old). That is because 1-2 days are required for the maturation of the female mosquito before a blood meal is taken, and then malaria parasites acquired by females from an infected blood meal require a lengthy period of development inside the mosquito before they can be transmitted. This period is known as the Extrinsic Incubation Period (EIP). Depending on the environmental temperature, this period is estimated to be about 9-15 days for *Anopheles *mosquitoes that transmit malaria parasites [8]. Therefore, *Anopheles *must be at least 11 days old (2 days after enclosure and 9 days for EIP) to be able to transmit malaria.

When age was considered as a binary variable (young; < 7 d old and old; ≥7 d old), binary logistic regression enabled a multivariate analysis of the effects of RNA*later*^® ^preservation, strain and actual age on age prediction accuracy. A clear decrease in the likelihood of an age misclassification was observed when mosquitoes were preserved in RNA*later*^® ^over fresh mosquitoes. The greatest effect on age prediction accuracy was the actual age of the mosquitoes for both datasets. Interpretation of the within-age effects was not straightforward; however, there was a tendency for mosquitoes with an actual age furthest from the cut-off age of 7 d to have a lower likelihood of a misclassification. As may be expected, the likelihood of a misclassification of age did not differ between strains.

Age and species identification in any mosquito vector control intervention is a vital success determinant of that intervention and thus plays an important part in vector control programmes. This and our previous studies on NIRS provide evidence to support the application of NIRS as a rapid assessment tool for vector control interventions targeting *An. gambiae s.s*. and *An. arabiensis *to measure relative species abundance and survival characteristics. NIRS age and species classification has great potential for evaluation of the epidemiology and control of mosquito borne diseases and the ability to work with preserved specimens enables this technique to be applied under difficult field conditions.

The results presented herein show age and species can be predicted from fresh mosquitoes with accuracies similar to those achieved from insects preserved in RNA*later*^®^. This dataset could be used to develop calibrations to predict age and species from lab-reared insects, but further work is needed to develop robust calibrations to include other sibling species, influences of physiological variations, wild mosquitoes, etc. These results were obtained from samples stored at -20°C, but no difference is expected if samples are stored at other temperatures or time intervals recommended by Ambion.

## Conclusions

In summary, RNA*later*^® ^can be used to preserve samples for subsequent age-grading and species identification by NIRS with reasonable confidence when compared to scans from fresh mosquitoes. This is a significant step forward as samples can have extended preservation time for later processing from the most challenging field locations. Costs associated with RNA*later*^® ^are estimated at 3-13 cents for a pool of 5 mosquitoes. Although an ideal situation would allow longer preservation at room temperature, the ability to stabilize samples *en route *from remote field locations is a significant step forward for the use of NIR technology. Additional work should focus on developing robust calibrations to predict the age and species of several other strains from diverse ecological settings.

## Competing interests

The authors declare that they have no competing interests.

## Authors' contributions

FED and KMD conceived the study; RAW, KM, SM, GFK, and FED designed the experiments; MS and KMD collected data; MS, KHSP, LH, GFK, and FED analysed the data; MS and FED drafted the manuscript. All authors reviewed and approved the final manuscript.
